# Solid‐Phase Conversion of Four Stereoisomers into a Single Enantiomer

**DOI:** 10.1002/anie.201808913

**Published:** 2018-10-19

**Authors:** Anthonius H. J. Engwerda, Johannes C. J. Mertens, Paul Tinnemans, Hugo Meekes, Floris P. J. T. Rutjes, Elias Vlieg

**Affiliations:** ^1^ Institute for Molecules and Materials Radboud University Heyendaalseweg 135 6525 AJ Nijmegen The Netherlands

**Keywords:** chirality, deracemization, diastereomers, racemization, Viedma ripening

## Abstract

Viedma ripening is an emerging method for the solid‐phase deracemization of mixtures of enantiomers. Up to now, the scope of the method has remained limited to molecules with a single stereocenter. We show here that this method can be extended to obtain a single enantiomer from a mixture of stereoisomers with two different stereocenters. In addition, we show that by using tailor‐made chiral additives, the conversion time can be reduced by a factor of 100.

Among the 45 drugs that were approved by the FDA (Food and Drug Administration) in 2015, thirteen were achiral, three contained a single stereocenter, sixteen were small molecules containing multiple stereocenters, and thirteen were large biomolecules.^1^ Due to the different bioactivity of the enantiomers, all of them (with the exception of the achiral drugs) are marketed as a single enantiomer. This clearly shows that obtaining molecules in enantiomerically pure form is of vital importance to human healthcare.^2^ When synthesis yields a combination of enantiomers, diastereomeric salt formation is a frequently used method to separate the desired enantiomer from its unwanted mirror image isomer.^3^ This implies, however, that half of the product is discarded. Alternatively, deracemization methods can be used, in which the unwanted enantiomer is converted into the desired product. One such deracemization method is Viedma ripening.^4^ This process involves the solid‐phase deracemization of a vigorously ground suspension of crystals, enabled by simultaneous solution‐phase racemization.^5^ A key requirement for the process is that the two enantiomers crystallize in separate crystals, that is, as a racemic conglomerate. Over the past few years, various types of molecules have been deracemized using this approach.^6^

Up to now, only molecules with a single stereocenter have been deracemized using Viedma ripening. However, as mentioned, the majority of drug molecules are enantiopure and contain multiple stereocenters. Conversion of such compounds into a single enantiomer using such grinding experiments is obviously more challenging. This is due to the process involving 2^*n*^ chiral compounds (with *n* the number of stereocenters), instead of only two. Of these 2^*n*^ compounds, one set of enantiomers will be the thermodynamically most favorable one, whereas the other diastereomers will have a higher energy (or higher solubility). Sakamoto et al. used this difference in stability for a related experiment.^7^ They studied a system with two stereocenters, of which one was enantiopure and could not epimerize, while the second one could epimerize in solution, resulting in a solid‐phase that contained only a single diastereomer. Other crystallization methods that lead to the formation of a single enantiomer exist as well.^8^ In the case of Viedma ripening, it is important that the thermodynamically more stable set of enantiomers crystallizes as a racemic conglomerate (Figure 1).

**Figure 1:**
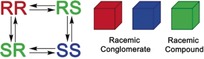
In order to successfully convert a compound with two stereocenters into a single stereoisomer using grinding experiments, epimerization of both stereocenters, as well as crystallization of the most stable pair of enantiomers as a racemic conglomerate, is required.

The crystallization behavior of the less stable diastereomers is expected to be less important, since these compounds will eventually be eliminated from the solid phase. More challenging is the interconversion (racemization) of the two enantiomers, since this requires epimerization of all chiral centers. This can be achieved if the centers epimerize in a (near) identical way, or if the conditions for the different epimerization pathways are compatible. As a first step towards multiple stereocenters, we herewith show a successful demonstration on two molecules with two different stereocenters to which these conditions apply. From a total of four diastereomers, a single one was obtained using grinding experiments. To the best of our knowledge, this is the first example of conversion of a stereoisomeric mixture of a compound with multiple stereocenters into only one enantiomer using such grinding experiments. So far, experiments by Hachiya et al. approximate this goal most closely, but using total spontaneous resolution instead.^9^ They succeeded in partially converting molecules with two identical stereocenters (meaning their system consisted of only three stereoisomers (one pair of enantiomers (**1**) and the corresponding achiral *meso* compound) instead of four stereoisomers (two pairs of enantiomers), Figure 2).

**Figure 2:**
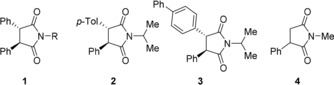
Deracemization of compounds of type **1** was previously studied by Hachiya et al.^12^ This study reports the conversion of compounds **2** and **3** into single enantiomers, both of which are structurally closely related to the anticonvulsant drug phensuximide (**4**).

Compounds **2** and **3** are examples of molecules with two different stereocenters, which thus exhibit four distinguishable stereoisomers. They belong to the class of succinimides, some of which exhibit anticonvulsant properties. They are structurally closely related to phensuximide (imide **4**), a drug used to treat epilepsy.^10^ Related methylated succinimides are known for their antifungal activity, of which the enantiopure (*R*,*R*)‐configured succinimides are the most effective ones.^11^ Obtaining such succinimides in enantiopure form is therefore highly relevant.

In case of compounds **2** and **3**, the *trans* diastereomers (3*R*,4*R* and 3*S*,4*S*) crystallize as a racemic conglomerate. For compound **2**, the *cis* isomers (3*R*,4*S* and 3*S*,4*R*) form a rare example of a solid solution.^12^ It is envisioned that the *trans* diastereomers are more stable, since this configuration is less sterically hindered. NMR experiments confirmed this hypothesis.

Epimerization of both stereocenters can be achieved by adding DBU (1,8‐diazabicyclo‐[5.4.0]undec‐7‐ene) as a base. When exposing a solution of compound **2** to epimerization conditions, only 2 % was present as the *cis* isomer at room temperature as observed in the ^1^H NMR spectrum. This corresponds to the conglomerate *trans* form being more stable by 2.5 kcal mol^−1^ (in solution), thus making this system potentially suitable for Viedma ripening (Figure 3).

**Figure 3:**
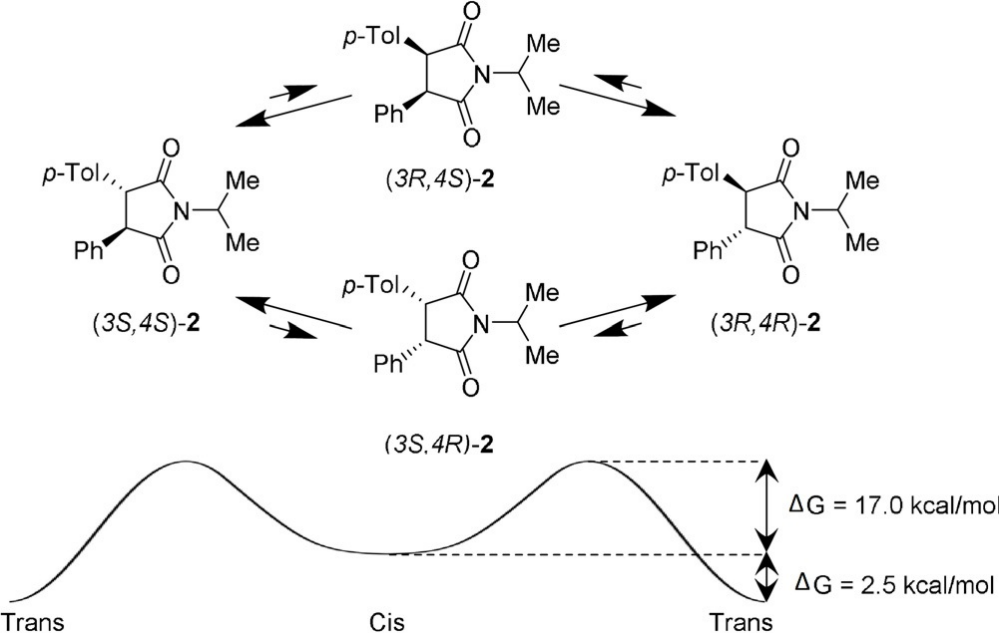
Epimerization and racemization of compound **2** takes place via reversible deprotonation of the stereocenters using DBU as a catalyst. In this case, the two epimerization rates are nearly identical. The indicated Gibbs free energies were determined using temperature‐dependent selective exchange spectroscopy.

Since the synthesis of compound **2** solely yielded the *cis* diastereomer (through hydrogenation of the corresponding maleimide derivative), this isomer was used as the starting point for the grinding experiments. Upon addition of the racemization catalyst (DBU), the dissolved *cis* diastereomers were epimerized into the *trans* form. Further dissolution and subsequent epimerization of *cis*‐**2** resulted in supersaturation and consequent crystallization of *trans*‐**2**. This process happened relatively fast in a time span of several minutes (Figure 4).

**Figure 4:**
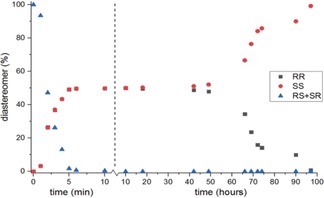
Example of a grinding experiment on a suspension of compound **2** starting from the *cis* diastereomers (quantities given are those of the solid state). During the first 10 minutes of the experiment, racemization was deliberately slowed down (by adding only a small fraction of the final amount of DBU) to show the conversion of the *cis* into the *trans* diastereomers (notice the two different time scales on the *x*‐axis). Note: the outcome of these experiments is stochastic, meaning that upon repeating the experiment, both enantiomers are equally likely to be obtained.

Using temperature‐dependent selective exchange spectroscopy measurements, it was found that the transition‐state barrier for the conversion of *cis* to *trans* was only Δ*G*=17.0 kcal mol^−1^. This results in a half‐life of approximately one second (using the Eyring equation). The limiting factor in this process is thus the dissolution, rather than the epimerization of the *cis* compound. Next, Viedma ripening conditions resulted in deracemization of the crystals to provide only one of the two *trans* enantiomers. Racemization of one *trans* isomer into the enantiomeric *trans* isomer happens at one center at a time, through sequential epimerization of the two chiral centers. The *cis* isomer is thus an intermediate in the racemization pathway (Figure 3). The racemization barrier of this process was calculated to be 19.5 kcal mol^−1^ (see the Supporting Information). The deracemization process took several days, showing the exponential behavior typical for Viedma ripening. The long deracemization time is largely caused by the long “dead time” before symmetry breaking occurs. Deracemization proceeds stochastically, meaning that different enantiomers are obtained as the crystalline product in different experiments. In similar grinding experiments, the production of a single enantiomer could also be achieved for a mixture of diastereoisomers of imide **3** (see Figure S3 in the Supporting Information).

Since it is synthetically more useful to be able to direct the outcome of a Viedma ripening experiment towards the desired enantiomer, we explored the possibility of using chiral additives, a strategy that was previously successfully applied to achieve this goal (alternatively, a small amount of enantiopure product might be added to achieve this same goal).^13^ A suitable chiral additive with high affinity for only one of the two crystal forms may act as a selective growth inhibitor.^14^ In practice, molecules that closely resemble one of the two enantiomers are selected for this purpose. Importantly, the chiral additive should not alter its stereochemical configuration during the experiment.

In this study, a combination of four enantiopure chiral additives was synthesized,^15^ all of which closely resemble the same enantiomer of compound **2** (Figure 5). All additives contain three chiral centers, of which one (carrying the hydroxy group) cannot epimerize under the influence of DBU. The locked chirality of this stereocenter also ensures the desired chirality of the other two centers (since epimerization of these centers would result in sterically unfavored *cis* orientations; see Supporting Information). Using these tailor‐made additives, the outcome of the grinding experiments always proceeded in the desired direction. The combination of the four additives based on (*R*,*R*)‐**2** always resulted in (*S*,*S*)‐**2** as the product, while (*S*,*S*)‐based additives resulted in (*R*,*R*)‐**2**. This phenomenon of opposing chirality has been extensively studied and is known as the Lahav rule of reversal.13b, 16 Since a combination of four additives was used in all experiments,^17^ we could not determine which of the four was most effective. The deracemization curve shows linear behavior, which is typical for the use of chiral additives.^18^ Not only could the desired enantiomer be obtained using this approach, but the time required for deracemization was also significantly reduced (Figure 6). When starting from the racemic *trans* diastereomers, the deracemization time was reduced from 70 to 2.5 hours. When starting from *cis*‐**2**, the conversion time was even reduced to less than one hour, in other words a reduction of almost a factor 100 was achieved.

**Figure 5:**
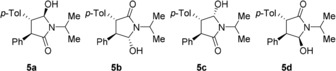
Additives for directing the outcome of the Viedma ripening experiments on compound **2**. In all cases, all four (in this case (*R*,*R*)‐based) additives were used together.

**Figure 6:**
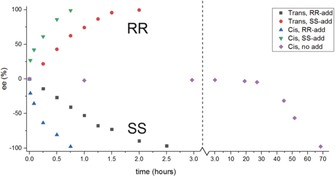
Curves showing the conversion of compound **2** into a single enantiomer, using 5 mol % of the chiral additives **5 a**–**d** (*ee* stands for enantiomeric excess; +100 % *ee* implies only *RR* while −100 % *ee* means only *SS*). Experiments were started from either the racemic *trans*‐(*R*,*R*/*S*,*S*) or *cis*‐(*R*,*S*/*S*,*R*) diastereomers. Within a minute after adding the racemization catalyst, all experiments contained only the *trans* diastereomer. When additives were used, **5 a**–**d** were always used as a combination. Please note the two different time scales on the *x*‐axis.

This increased conversion speed is induced in the initial phase of the process. Fast epimerization of the *cis* diastereomer results in a supersaturated solution of the *trans* enantiomers. Since the rate of *cis*‐to‐*trans* epimerization is higher than that of racemization, this results in crystallization of both *trans* enantiomers in the absence of an additive. In the presence of the additive, however, crystallization of the enantiomer similar to the additive is hampered, thereby resulting in preferential crystallization of the other enantiomer.^19^ This results in a solid phase that already has a high starting *ee* (around 25 % after adding the racemization catalyst) when the Viedma ripening commences. The time required to obtain a single enantiomer is therefore even shorter than with the use of an additive starting from *trans*‐**2**.

In conclusion, we have shown that the formation of one single isomer out of four stereoisomers using isomerization and subsequent Viedma ripening is possible. The requirements are similar to those for compounds with only a single stereocenter but can in practice be difficult. The desired pair of enantiomers should be the thermodynamically most stable pair and should also crystallize as a racemic conglomerate. However, when the most stable pair of enantiomers crystallizes as a racemic compound, derivatization^20^ or salt formation^21^ can additionally be used to convert it into a conglomerate. Racemization can be more challenging, since epimerization of all chiral centers should proceed under the same conditions. These principles hold not only for a molecule with two stereocenters, but also for more stereocenters. As long as these requirements are met, Viedma ripening may be used for the deracemization of any compound with multiple stereocenters.

## Conflict of interest

The authors declare no conflict of interest.

## Supporting information

As a service to our authors and readers, this journal provides supporting information supplied by the authors. Such materials are peer reviewed and may be re‐organized for online delivery, but are not copy‐edited or typeset. Technical support issues arising from supporting information (other than missing files) should be addressed to the authors.

Supplementary
