# Supplementary material for: Solid‐Phase Conversion of Four Stereoisomers into a Single Enantiomer
Source: Angew Chem Int Ed Engl. 2018 Oct 19;57(47):15441–4. doi: 10.1002/anie.201808913 (PMC6282611; doi:10.1002/anie.201808913)
Supplement: Supplementary file 1 — Supplementary [file ANIE-57-15441-s001.pdf]

Angewandte Chemie
Eine Zeitschrift der Gesellschaft Deutscher Chemiker

Supporting Information

Solid-Phase Conversion of Four Stereoisomers into a Single Enantiomer

Anthonius H. J. Engwerda, Johannes C. J. Mertens, Paul Tinnemans, Hugo Meekes, Floris P. J. T. Rutjes, and Elias Vlieg**

anie_201808913_sm_miscellaneous_information.pdf

Supporting Information

Table of contents: **Page:**

1. Synthesis of diastereomers and additives	2
2. Temperature-dependent NMR experiments	6
3. Deracemization experiments	8
4. Powder diffractograms	10
5. CD-spectra	11
6. HPLC chromatograms	12
8. Determination of absolute configuration	17
9. NMR spectra	18
10. References	22

1 Synthesis of diastereomers and additives

General:
All commercial chemicals were purchased from Sigma-Aldrich and were used without further purification. Reactions were followed using thin layer chromatography (TLC) on silica gel-coated plates (Merck 60 F254). Detection was performed with UV-light (254 nm), and/or by charring at ~150 °C after dipping into a solution of $KMnO_4$ (1 g/100 mL) in ethanol. NMR spectra were recorded on a Varian 400 (400 MHz) spectrometer in $CDCl_3$ (unless otherwise reported). Chemical shifts are given in ppm with respect to tetramethylsilane (TMS) as internal standard. Coupling constants are reported as *J*-values in Hz. HPLC analysis was performed on a chiral HPLC (ADH column, isocratic, 10% IPA in heptane, flow 1 mL min^{-1} UV detection, 215 nm).

Compound **2** was synthesized starting from 4-methylphenylacetic acid and phenylglyoxylic acid in three steps, based on the procedure of Hachiya et al.[1]. In contrast to their procedure, Pd/C was used as a hydrogenation catalyst instead of PtO_2.

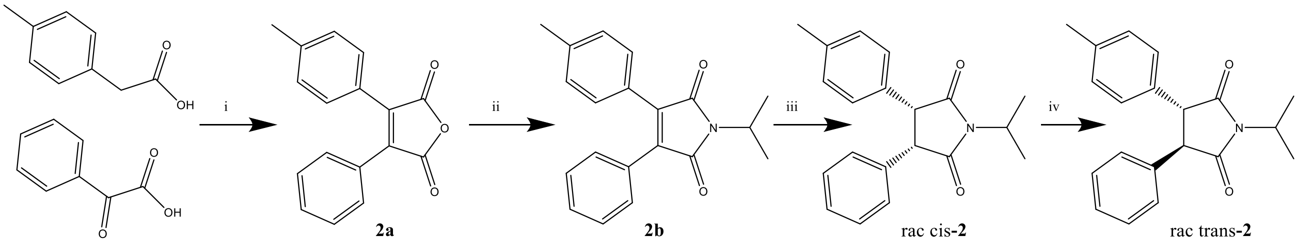

*3-Phenyl-4-(4-methylphenyl)maleic anhydride (**2a**):* 4-Methylphenylacetic acid (10.4 g, 69.2 mmol) and phenylglyoxylic acid (10.4 g, 69.2 mmol) were suspended in acetic anhydride (50 mL). The mixture was refluxed for 3 h, quenched with an excess of water and filtered. The residue was triturated with acetone, the filtrate of which was evaporated *in vacuo*. Recrystallization from ethanol and drying *in vacuo* yielded yellow crystals (10.9 g, 41.4 mmol). The recrystallization filtrate was recrystallized from ethanol and water to obtain a second batch of yellow crystals (2.7 g, 10.1 mmol, combined yield 74%). ^{1}H NMR (400 MHz, $CDCl_3$) δ 7.58-7.53 ($C\underline{H}_{arom}$, m, 2H), 7.50-7.39 ($C\underline{H}_{arom}$, m, 5H), 7.12-47.19 ($C\underline{H}_{arom}$, m, 2H), 2.40 ($C\underline{H}_3$, s, 3H). ^{13}C NMR (100 MHz, $CDCl_3$) δ 165.0, 164.9, 141.9, 138.2, 137.2, 130.9, 129.7, 129.6, 129.6, 128.9, 127.4, 124.3, 21.6.

*N-Isopropyl-3-phenyl-4-(4-methylphenyl)maleimide **2b**:* Compound **2a** (7.95 g, 30.1 mmol) was dissolved in toluene–DMF (3:1 v/v, 25 mL) and cooled to 0 °C. Isopropylamine (25 mL, 291 mmol) was added and the mixture was stirred for 1.5 h, after which excess isopropylamine was removed *in vacuo*. The mixture was refluxed for 4 h and subsequently evaporated and dried *in vacuo* to obtain yellow crystals (9.82 g, 32.2 mmol, quant.).^{1}H NMR (400 MHz, $CDCl_3$) δ 7.50-7.44 ($C\underline{H}_{arom}$, m, 2H), 7.40-7.31 ($C\underline{H}_{arom}$, m, 5H), 7.17-7.12 ($C\underline{H}_{arom}$, m, 2H), 4.48 ($C\underline{H}(CH_3)_2$, septet, 1H, *J* = 6.9 Hz), 2.36 ($C\underline{H}_3$, s, 3H), 1.48 ($C\underline{H}_3$, d, 6H, *J* = 7.1Hz). ^{13}C NMR (100 MHz, $CDCl_3$) δ 170.8, 170.8, 140.0, 136.0, 135.2, 129.8, 129.8, 129.5, 129.2, 129.0, 128.5, 125.8, 43.3, 21.5, 20.2.

*cis-1-Isopropyl-3-phenyl-4-(4-methylphenyl)pyrrolidine-2,5-dione (R,S/S,R-**2**):* Compound **b** (12.0 g, 39 mmol) was dissolved in 20 mL ethyl acetate. After adding 500 mg Pd/C (10%), the suspension was stirred under 30 bar hydrogen pressure for 24 hours. After completion of the reaction (monitored by the change of a yellow to a colorless solution), the suspension was filtered over Celite to remove the Pd/C. After removal of the solvent, seed crystals were added to induce crystallization, after which *cis*-**2** was obtained as a colorless solid (initial crystals were obtained by pouring liquid nitrogen on the oil of *cis*-**2**). ^{1}H NMR (400 MHz, $CDCl_3$) δ 7.11-7.05 (C$\underline{H}_{arom}$, m, 3H), 6.90-6.82 (C$\underline{H}_{arom}$, m, 4H), 6.72-6.68 (C$\underline{H}_{arom}$, m, 2H), 4.65 (C$\underline{H}$(CH_3)$_2$, septet, 1H, *J* = 7.0 Hz), 4.43-4.36 (C$\underline{H}$, m, 2H), 2.16 (C$\underline{H}_3$, s, 3H), 1.57 (C$\underline{H}_3$, d, 6H, *J* = 7.0 Hz). ^{13}C NMR (100 MHz, $CDCl_3$) δ 177.1, 177.2, 136.8, 134.2, 131.0, 129.2, 129.0, 128.9, 128.2, 127.2, 51.9, 52.2, 44.2, 21.0, 19.5.

*rac-trans-1-Isopropyl-3-phenyl-4-(4-methylphenyl)pyrrolidine-2,5-dione (R,R/S,S-**2**):* The *trans* diastereomers could be readily prepared by dissolving *cis*-**2** in chloroform and adding catalytic amounts (2 mol%) of DBU. After stirring for one hour, the solvent was partially removed *in vacuo* and heptane was added, resulting in the crystallization of *trans*-**2** as colorless crystals. ^{1}H NMR (400 MHz, $CDCl_3$) δ 7.41-7.29 (C$\underline{H}_{arom}$, m, 3H), 7.21-7.15 (C$\underline{H}_{arom}$, m, 4H), 7.10-7.05 (C$\underline{H}_{arom}$, m, 2H), 4.55 (C$\underline{H}$(CH_3)$_2$, septet, 1H, *J* = 6.9 Hz), 3.98-3.92 (C$\underline{H}$, m, 2H), 2.35 (C$\underline{H}_3$, s, 3H), 1.57 (C$\underline{H}_3$, appears as a triplet, 6H, *J* = 7.1Hz). ^{13}C NMR (100 MHz, $CDCl_3$) δ 176.6, 176.7, 137.6, 137.0, 133.9, 129.7, 129.0, 127.7, 127.4, 127.2, 55.1, 54.8, 44.2, 20.9, 19.3, 19.1.

Compound **3** was synthesized, using a similar procedure as for the synthesis of **2**, starting from 4-bilphenylacetic acid and phenylglyoxylic acid.

*3-Phenyl-4-(4-biphenyl)maleic anhydride (**3a**):* 4-Biphenylacetic acid (10.6 g, 50.0 mmol) and phenylglyoxylic acid (7.5 g, 50.0 mmol) were suspended in acetic anhydride (50 mL). The mixture was refluxed for 3 h, quenched with an excess of water and filtered. The residue was triturated with acetone, the filtrate of which was evaporated *in vacuo*. The obtained yellow solid was used in the next step without any further purification.

*N-Isopropyl-3-phenyl-4-(4-biphenyl)maleimide **3b**:* Compound **3a** was dissolved in toluene–DMF (3:1 v/v, 25 mL) and cooled to 0 °C. Isopropylamine (25 mL, 291 mmol) was added and the mixture was stirred for 1.5 h, after which excess isopropylamine was removed *in vacuo*. The mixture was refluxed for 4 h and subsequently evaporated and dried *in vacuo* to obtain yellow crystals (10.3 g, 27.9 mmol, 56% over two steps). ^{1}H NMR (400 MHz, $CDCl_3$) δ 7.64-7.35 (C$\underline{H}_{arom}$, m, 14H), 4.52 (C$\underline{H}$(CH_3)$_2$, septet, 1H, *J* = 7.1 Hz), 1.51 (C$\underline{H}_3$, d, 6H, *J* = 6.8Hz). ^{13}C NMR (100 MHz, $CDCl_3$) δ 170.7, 170.6, 142.4, 140.2, 135.7, 135.5, 130.4, 129.9, 129.8, 129.7, 128.8, 128.5, 127.8, 127.6, 127.2, 127.1, 43.4, 20.2

*cis-1-Isopropyl-3-phenyl-4-(4-biphenyl)pyrrolidine-2,5-dione (R,S/S,R-**3**):* Compound **3b** (10.3 g, 27.9 mmol) was dissolved in 20 mL ethyl acetate. After adding 500 mg Pd/C (10%), the suspension was stirred under 30 bar hydrogen pressure for 24 hours. After completion of the reaction (monitored by the change of a yellow to a colorless solution), the suspension was filtered over Celite to remove the Pd/C. After removal of the solvent, seed crystals were added to induce crystallization, after which *cis*-**3** was obtained

as a colorless solid (initial crystals were obtained by pouring liquid nitrogen on the oil of *cis*-**3**). In contrast to the synthesis of cis-**2**, which yielded only the trans-diastereomer, the solid contained a 8:1 ratio of cis:trans-**3**. ^{1}H NMR (400 MHz, $CDCl_3$) δ 7.11-7.05 (CH_{arom}, m, 3H), 6.90-6.82 (CH_{arom}, m, 4H), 6.72-6.68 (CH_{arom}, m, 2H), 4.65 ($CH(CH_3)_2$, septet, 1H, *J* = 7.0 Hz), 4.43-4.36 (CH, m, 2H), 1.59 (CH_3, d, 6H, *J* = 7.1 Hz). ^{13}C NMR (100 MHz, $CDCl_3$) δ 177.1, 177.1, 140.0, 133.0, 129.8, 129.6, 129.1, 128.7, 128.3, 127.4, 127.3, 127.1, 126.9, 126.8, 52.3, 21.9, 44.4, 19.5, 19.4

*rac-trans-1-Isopropyl-3-phenyl-4-(4-biphenyl)pyrrolidine-2,5-dione (R,R/S,S-**3**):* The *trans*-diastereomers could be readily prepared by dissolving *cis*-**3** in chloroform and adding catalytic amounts (2 mol%) of DBU. After stirring for one hour, the solvent was partially removed *in vacuo* and heptane was added, resulting in the crystallization of *trans*-**3** as colorless crystals. ^{1}H NMR (400 MHz, $CDCl_3$) δ 7.64-7.29 (CH_{arom}, m, 7H), 7.11-7.04 (CH_{arom}, m, 3H), 6.91-6.84 (CH_{arom}, m, 4H), 4.67 ($CH(CH_3)_2$, septet, 1H, *J* = 7.0 Hz), 4.05 (CH, s, 2H), 1.53 (CH_3, appears as a triplet, 6H, *J* = 6.8 Hz). ^{13}C NMR (100 MHz, $CDCl_3$) δ 176.7, 176.6, 140.9, 140.4, 136.9, 135.8, 129.2, 128.8, 128.0, 127.9, 127.9, 127.5, 127.4, 127.0, 55.2, 55.0, 44.5, 19.5, 19.3

Synthesis of additives **5a**-**d**:

The synthesis of chiral additives **5a**-**d** was based on the procedure described by Wijnberg et al.[2] Enantiopure compound **2** (0.5 g, 1.6 mmol) and $CeCl_3.7H_2O$ (0.6 g, 1.6 mmol) were added to 10 mL ethanol. The stirred solution was cooled on ice, after which 2 g $NaBH_4$ (50 mmol, 31 eq) was added over a period of two hours. After an additional 4 hours of stirring at 0°C, water was added to quench the reaction. The product was extracted using diethyl ether and was dried with Na_2SO_4. After removal of the solvent, a 5:5:1:1 mixture of **5a**:**5b**:**5c**:**5d** was obtained in quantitative yield. All additives were obtained in enantiopure form, as could be derived from the comparison of the HPLC chromatograms of enantiopure and racemic products. To test whether the additives were resistant to racemization, a solution of the enantiopure additives in chloroform was exposed to 1 equivalent of DBU for 4 days. After this time, the additives remained enantiopure, according to chiral HPLC.

NMR spectra of **5a** + **5b (major):**

No distinction could be made between the NMR spectra of compounds **5a** and **5b**. For the ^{1}H spectrum, most peaks overlapped (with the exception of both CH_3 peaks). Assignment of the ^{1}H peaks to the corresponding protons was done based on COSY, HMBC and HSQC NMR experiments. For the ^{13}C spectrum, two separate peaks could be attributed to the combination of the two species. The chemical shifts of both peaks are given. ^{1}H NMR (400 MHz, $CDCl_3$) δ 7.36-7.03 (CH_{arom}, m, 9H), 5.22 (CHOH, ddd, *J* = 7.4, 5.1, 3.5 Hz), 4.34 ($CH(CH_3)_2$, two septets, separated by 1.6 Hz, *J* = 6.6 Hz), 3.68 (COCH, dd (appears as a triplet), *J* = 7.0, 7.0 Hz),), 3.26 (CHCHOH, ddd, *J* = 10.6, 6.6, 3.5 Hz), 2.96 (dd, *J* = 12.5, 7.4 Hz), 2.33 (s, two peaks separated by 13.7 Hz), 1.35 (two doublets, separated by 17.6 Hz, *J* = 7.0 Hz). ^{13}C NMR (100 MHz, $CDCl_3$) δ 173.3, 173.2, 140.7, 139.0, 137.7, 137.0, 136.7, 135.9, 129.7, 129.5, 128.9, 128.0, 127.8, 127.0, 126.9, 87.6, 87.5, 57.3, 56.9, 55.9, 55.4, 44.4, 22.3, 21.1, 21.0, 19.2.

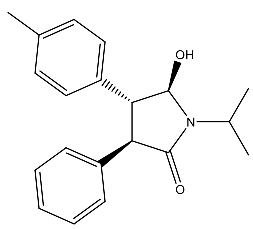

5a

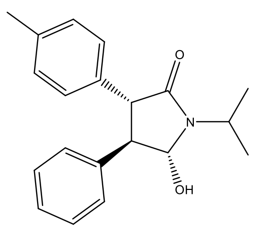

5b

NMR spectra of **5c** + **5d (minor):**

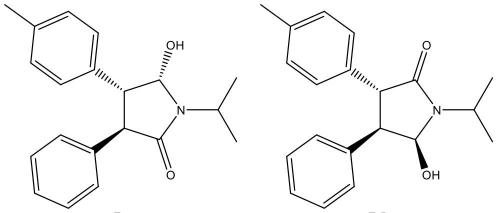

5c 5d

No distinction could be made between the NMR spectra of compounds **5c** and **5d**. For the ^{1}H spectrum, all peaks overlapped (with the exception of the CH_3 peaks). Assignment of the ^{1}H peaks to the corresponding protons was done based on COSY, HMBC and HSQC NMR experiments. For the ^{13}C spectrum, two separate peaks could be attributed to the combination of the two species. The chemical shifts of both peaks are given. ^{1}H NMR (400 MHz, $CDCl_3$) δ 7.36-7.03 (C$\underline{H}_{arom}$, m, 9H), 5.39 (ddd, *J* = 9.0, 5.1, 3.9Hz), 4.34 (two septets, separated by 1.6 Hz, *J* = 6.6 Hz), 4.18 (dd, *J* = 10.9, 3.5 Hz), 3.60 (ddd, *J* = 11.3, 7.4, 5.1 Hz), 2.29 (s, two peaks separated by 24.2 Hz), 2.01 Hz, (dd, *J* = 5.9, 3.9 Hz), 1.35 (two doublets, separated by 17.6 Hz, *J* = 7.0 Hz). ^{13}C NMR (100 MHz, $CDCl_3$) δ 174.6, 174.5, 137.5, 137.1, 136.7, 135.5, 133.9, 132.2, 129.6, 129.2, 128.8, 128.7, 128.5, 128.4, 128.3, 127.3, 127.1, 81.0, 81.1, 53.5, 53.8, 50.2, 49.8, 44.2, 44.2, 21.9, 21.8, 21.0, 20.1.

2 Temperature-dependent NMR experiments

Calculation of the thermodynamic parameters of the racemization of compound **2**:

In these experiments, *trans*-**2** (0.1 g, 0.3 mmol) and DBU (2 µL, 0.013 mmol, 5 mol%) were dissolved in 0.8 mL deuterated chloroform. Then, the ^{1}H NMR spectrum was recorded at several temperatures. At all these temperatures, the percentage *cis* diastereomer was determined, from which the equilibrium constant (*K*) could be calculated. Using the Van ’t Hoff equation, the thermodynamic parameters of the equilibrium between *cis* and *trans* could be calculated:

$$\ln(K) = -\frac{\Delta H}{RT} + \frac{\Delta S}{R}$$

Δ*H* = 2.8 kcal/mol, Δ*S* = 1.2 cal/molK, Δ*G* = 2.5 kcal/mol (at RT)

In order to determine the energy barrier of racemization, temperature-dependent selective Exchange Spectroscopy (EXSY) experiments were performed. In these experiments, the NMR peaks of the protons at the chiral center of *cis*-**2** were excited and conversion into *trans*-**2** was observed after several mixing (waiting) times (figure S1). The rate constant of this process was determined at various temperatures (figure S1), from which the Eyring plot could be constructed (figure S2). Using the Eyring plot, the entropy, enthalpy and Gibbs free energy of the transition state could be determined:

$$\ln\left(\frac{k}{T}\right) = -\frac{\Delta H}{R} * \frac{1}{T} + \ln\left(\frac{k_b}{h}\right) + \frac{\Delta S}{R}$$

Δ*H* = 5.2 kcal/mol, Δ*S* = -168 cal/molK, Δ*G* = 17.0 kcal/mol (at RT)

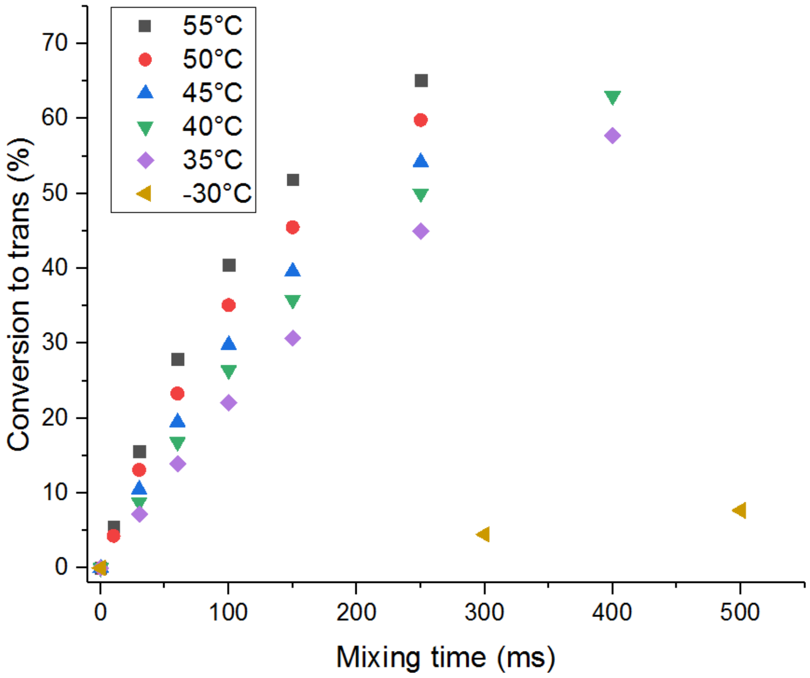

Figure S1: Conversion of *cis*-**2** into *trans*-**2** determined using temperature-dependent selective Exchange Spectroscopy Experiments.

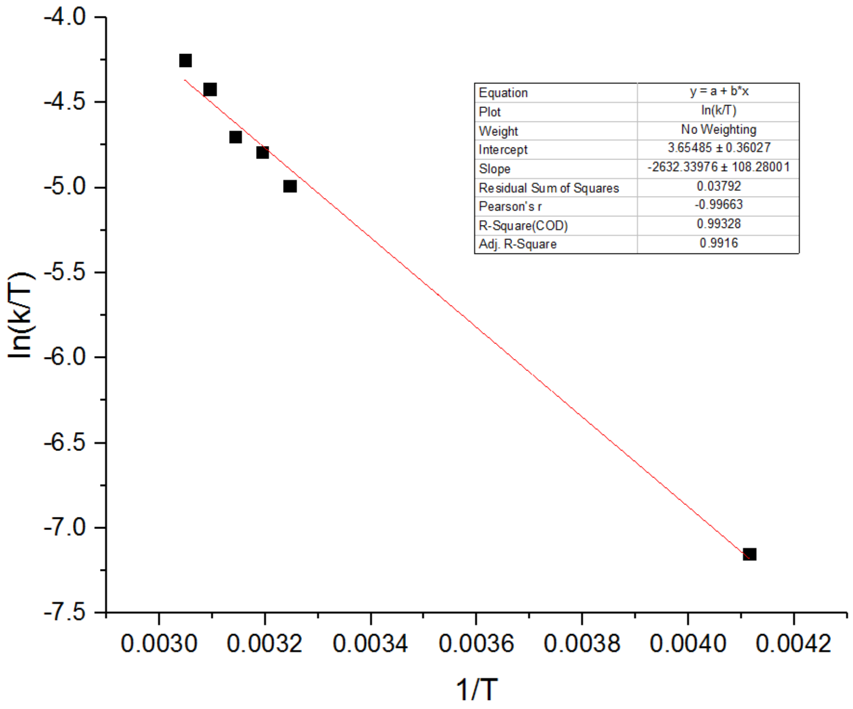

Figure S2: Eyring plot of the rate constant of the conversion of *cis* into *trans*-**2**.

3 Deracemization experiments

Deracemization experiments starting from *cis*-RS+SR-**2**:
Cis-**2** (800 mg), glass beads (5 g, Ø ca. 2 mm VWR international) and an oval PTFE-coated magnetic stirring bar (L 20 mm, Ø 10 mm) were added to 7 mL heptane and 0.8 mL chloroform in a 15 mL vial. The suspension was stirred at 600 rpm for 1 hour to ensure homogenization of the crystal sizes. Next, 1 µL DBU was added. During the next 320 minutes samples were taken regularly to observe the conversion of the RS+SR into the RR+SS diastereomers. Another 30 µL DBU was added, after which grinding was continued.

Deracemization experiments using chiral additives:
Cis- or *trans*-**2** (450 mg), glass beads (4 g, Ø ca. 2 mm VWR international) and an oval PTFE-coated magnetic stirring bar (L 20 mm, Ø 10 mm) were added to 4 mL heptane and 0.5 mL chloroform in a 15 mL vial. When additives were used, a total of 20 mg enantiopure additive was added as well. The suspensions were stirred at 600 rpm for 1 hour to ensure homogenization of the crystal sizes. Next, 20 µL DBU was added, after which samples were taken regularly.

Sampling:
For sampling, 100 µL of the suspension was taken using a syringe. The crystals were filtered off on a P4 glass filter and were washed with 0.1 mL diethyl ether to remove the remaining DBU.

Deracemization experiments of-**3**:

Compound-**3** (800 mg), glass beads (5 g, Ø ca. 2 mm VWR international) and an oval PTFE-coated magnetic stirring bar (L 20 mm, Ø 10 mm) were added to 6 mL heptane and 3 mL chloroform in a 15 mL vial. The suspension was stirred at 600 rpm for 1 hour to ensure homogenization of the crystal sizes. Next, 30 µL DBU was added, after which grinding was continued. Samples were taken regularly during the experiment.

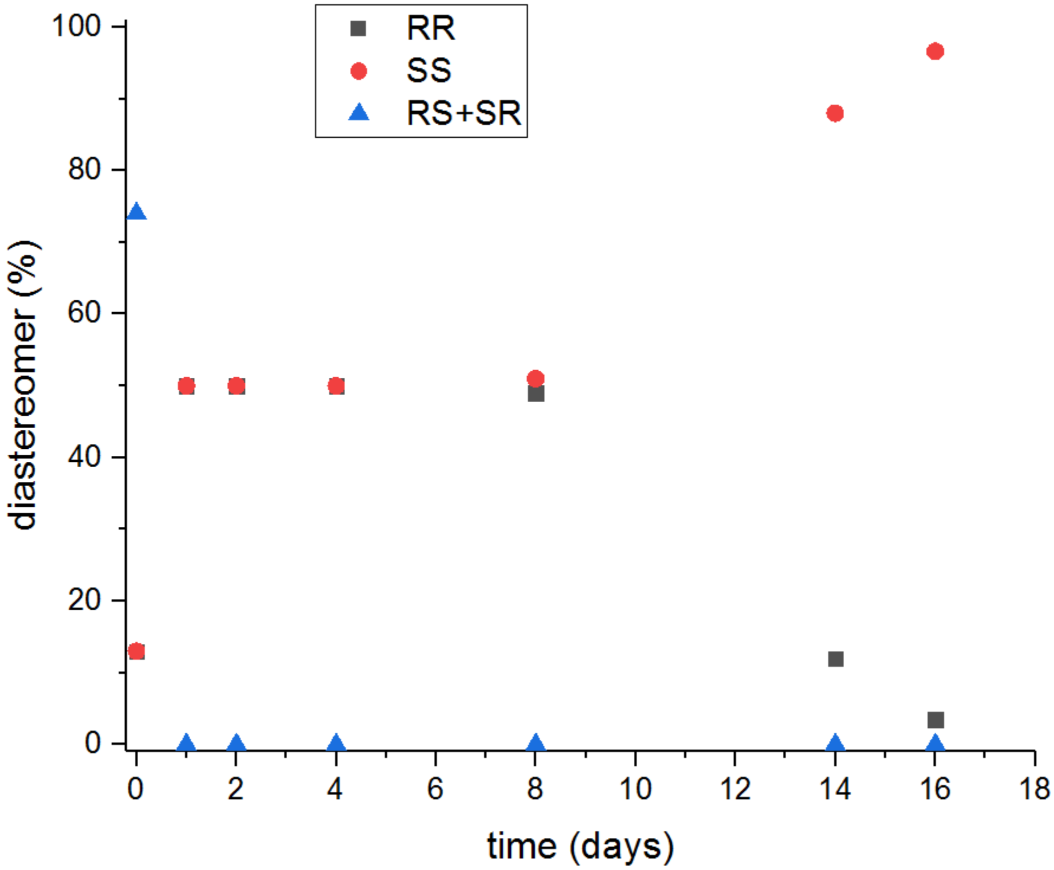

Figure S3: Deracemization curve of compound **3**.

4 Powder Diffractograms

All diffractograms were recorded using a Bruker D8 Advance Spectrometer using a VANTEX detector. Data was recorded using reflection mode with monochromatic Cu-Kα_1 radiation. The patterns based on the crystal structures were corrected for the different temperature at which they were recorded.

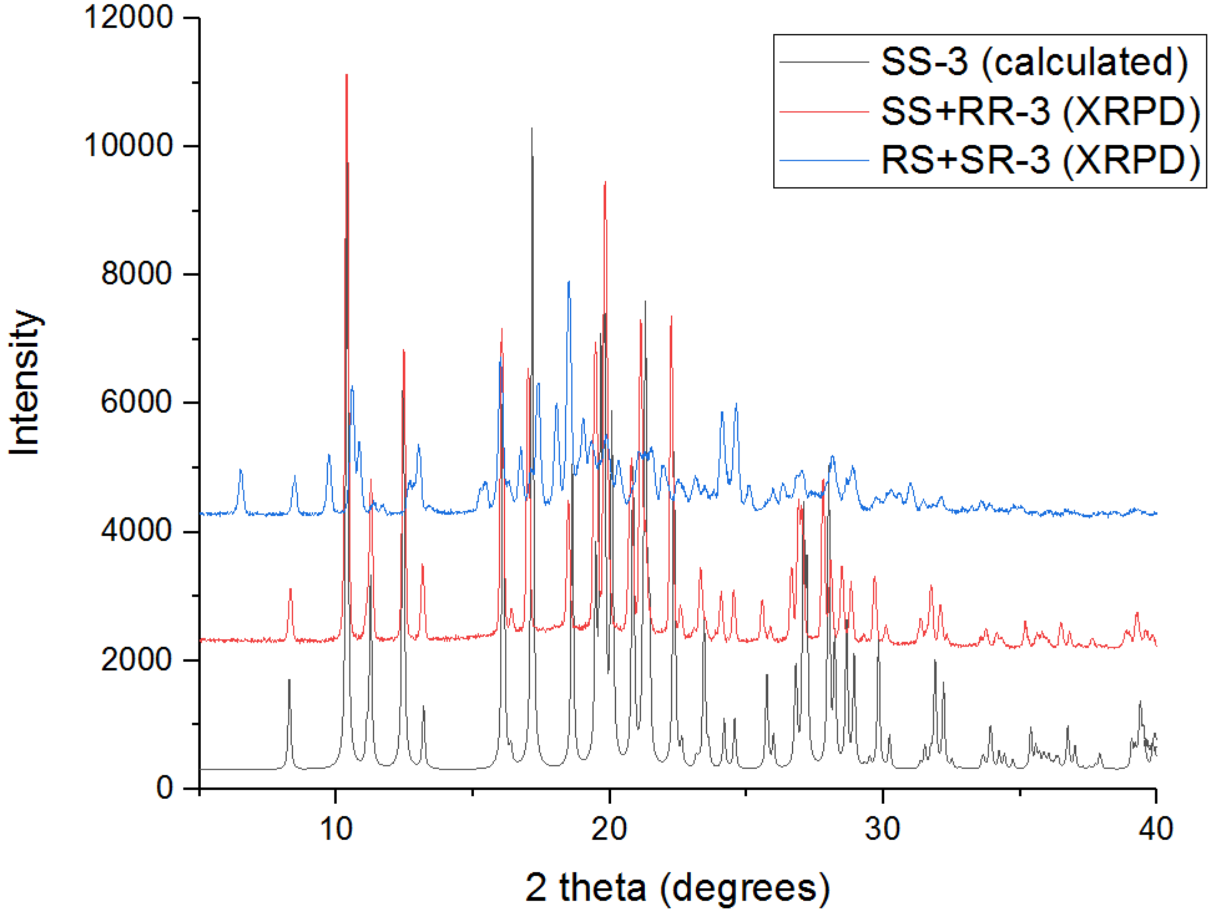

Figure S4: Powder diffractograms of the diastereomers of compound **3**. For SS-**3**, the expected pattern based on the newly reported crystal structure is also given.

5 CD Spectra

CD spectra were recorded on a JASCO J-815 CD spectrometer. Samples were dissolved (approximately 1mg/mL) in ethanol.

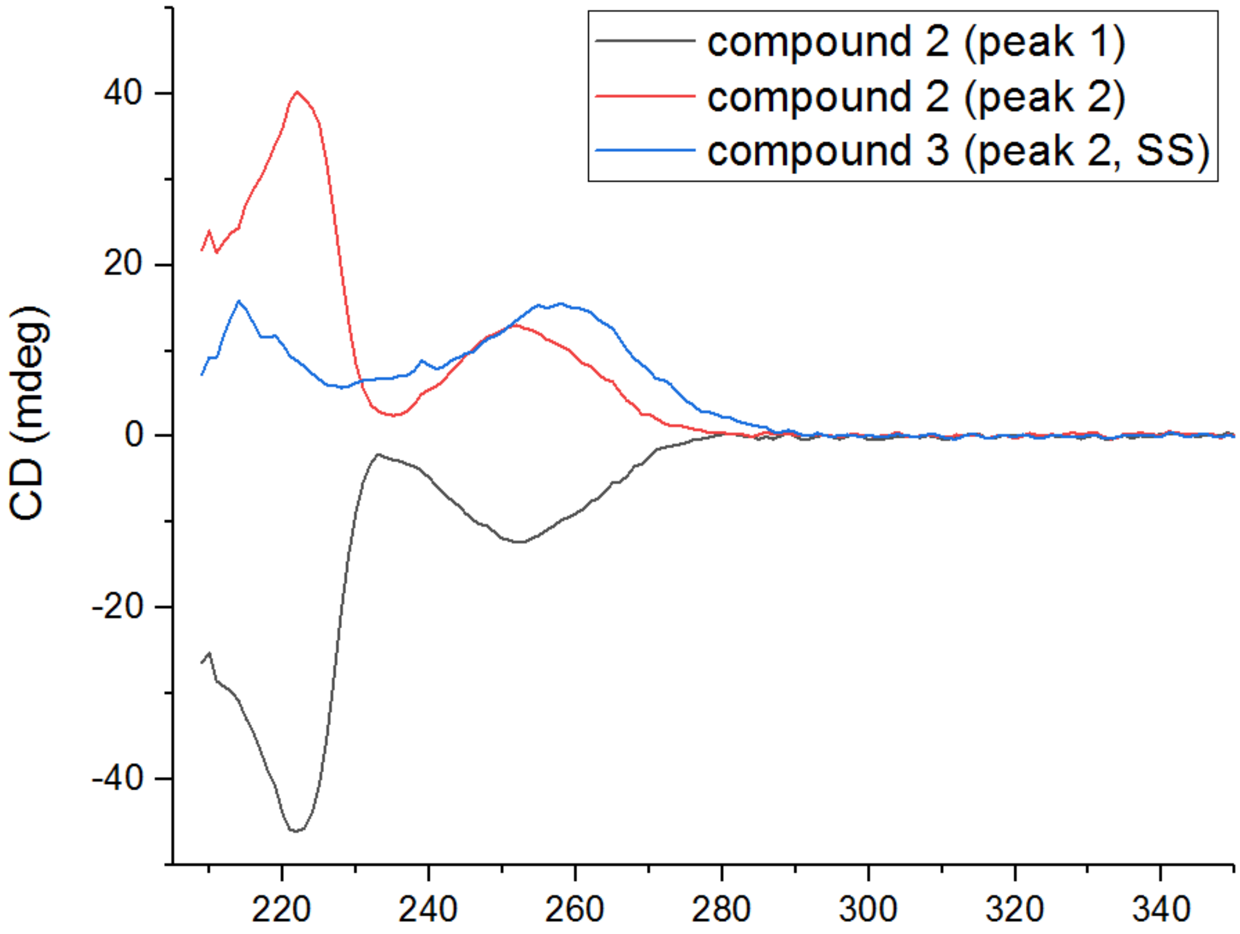

Figure S5: CD spectrums of compounds **2** and **3**.

6 HPLC chromatograms

The *ee* of all samples of compound **2** was determined using chiral HPLC (ADH column, 10% IPA in heptane, flow 1 mL min^{-1}).

Chromatogram of RS+SR-**2**

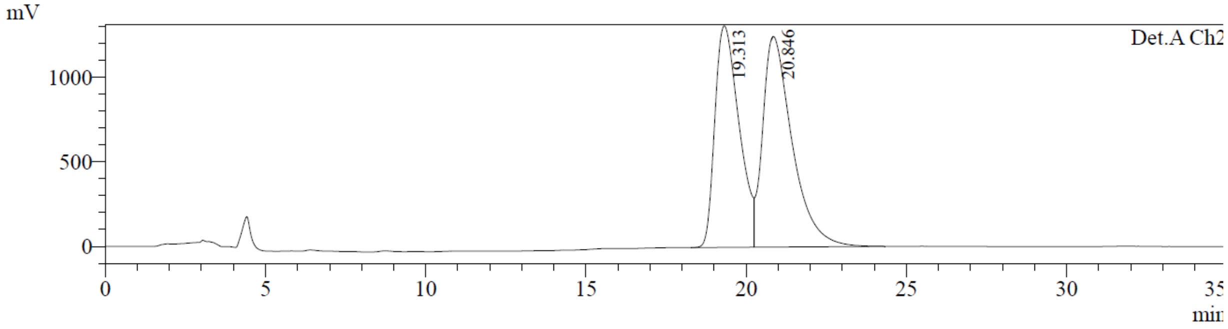

Detector A Ch2 215nm

Peak#	Ret. Time	Area	Height	Area %	Height %
1	19.313	68847671	1310538	46.354	51.270
2	20.846	79677081	1245594	53.646	48.730
Total		148524753	2556132	100.000	100.000

Chromatogram of RR+SS-**2**

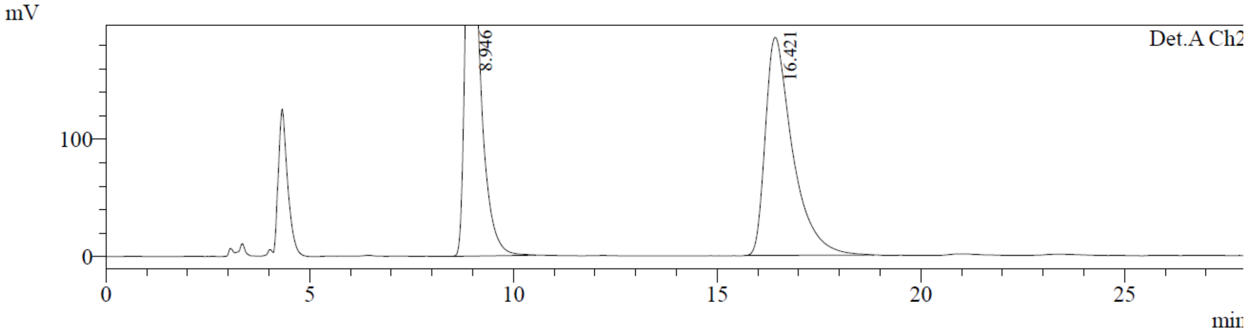

Detector A Ch2 215nm

Peak#	Ret. Time	Area	Height	Area %	Height %
1	8.946	8627872	352653	49.841	65.461
2	16.421	8682790	186072	50.159	34.539
Total		17310662	538724	100.000	100.000

Chromatogram of SS-**2**

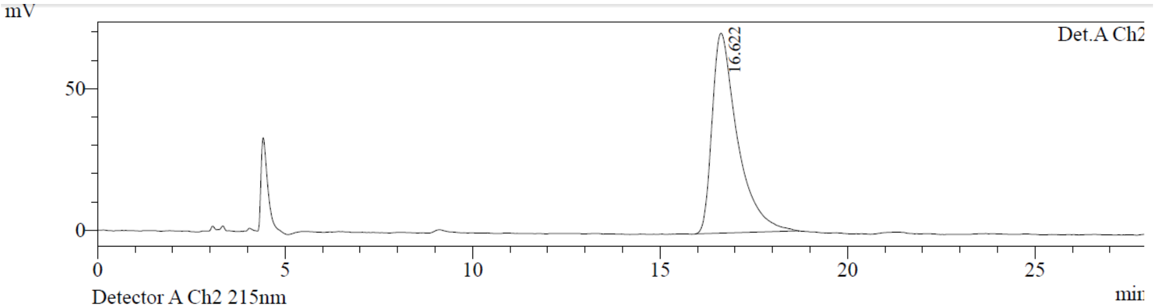

Detector A Ch2 215nm

Peak#	Ret. Time	Area	Height	Area %	Height %
1	16.622	3325932	70680	100.000	100.000
Total		3325932	70680	100.000	100.000

Chromatogram of RS+SR+RR+SS-**2**

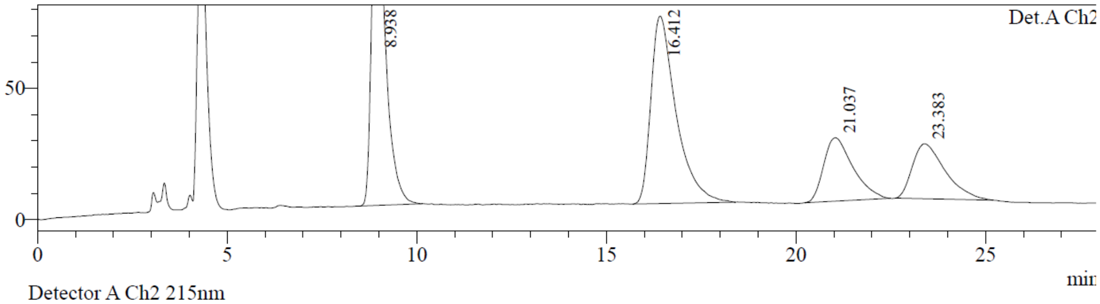

Detector A Ch2 215nm

Peak#	Ret. Time	Area	Height	Area %	Height %
1	8.938	3276073	135190	35.830	53.724
2	16.412	3303466	71372	36.129	28.363
3	21.037	1299924	24142	14.217	9.594
4	23.383	1263946	20936	13.824	8.320
Total		9143410	251640	100.000	100.000

The *ee* of all samples of compound **3** was determined using chiral HPLC (ADH column, 20% IPA in heptane, flow 1 mL min^{-1}).

Chromatogram of SS+RR-**3**

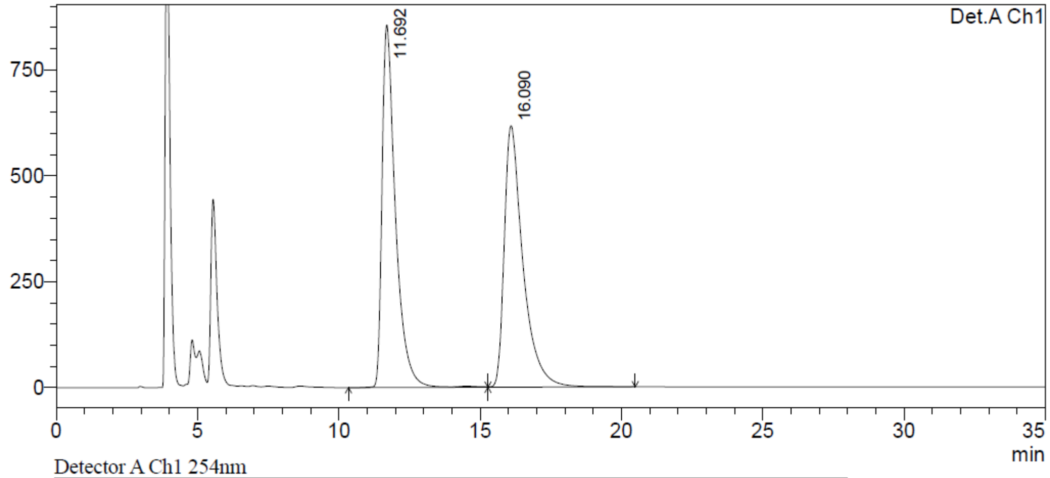

Detector A Ch1 254nm

Peak#	Ret. Time	Area	Height	Area %	Height %
1	11.692	28312078	855815	49.952	58.095
2	16.090	28366611	617316	50.048	41.905
Total		56678689	1473131	100.000	100.000

Chromatogram of a single crystal of SS-**3** (note: the absolute configuration of the crystal was determined using single-crystal X-ray diffraction)

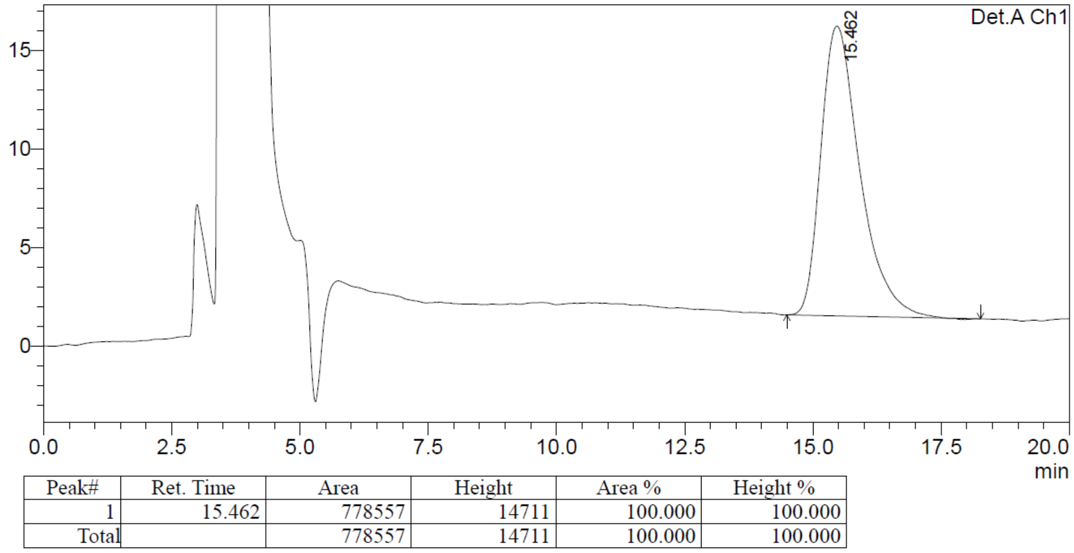

Peak#	Ret. Time	Area	Height	Area %	Height %
1	15.462	778557	14711	100.000	100.000
Total		778557	14711	100.000	100.000

Chromatogram of a single SS+RR+SR+RS -**3** (note: the *cis*-**3** to *trans*-**3** ratio is approximately 4:1 for this chromatogram. Also one of the *cis*-peaks overlaps with one of the *trans*-peaks)

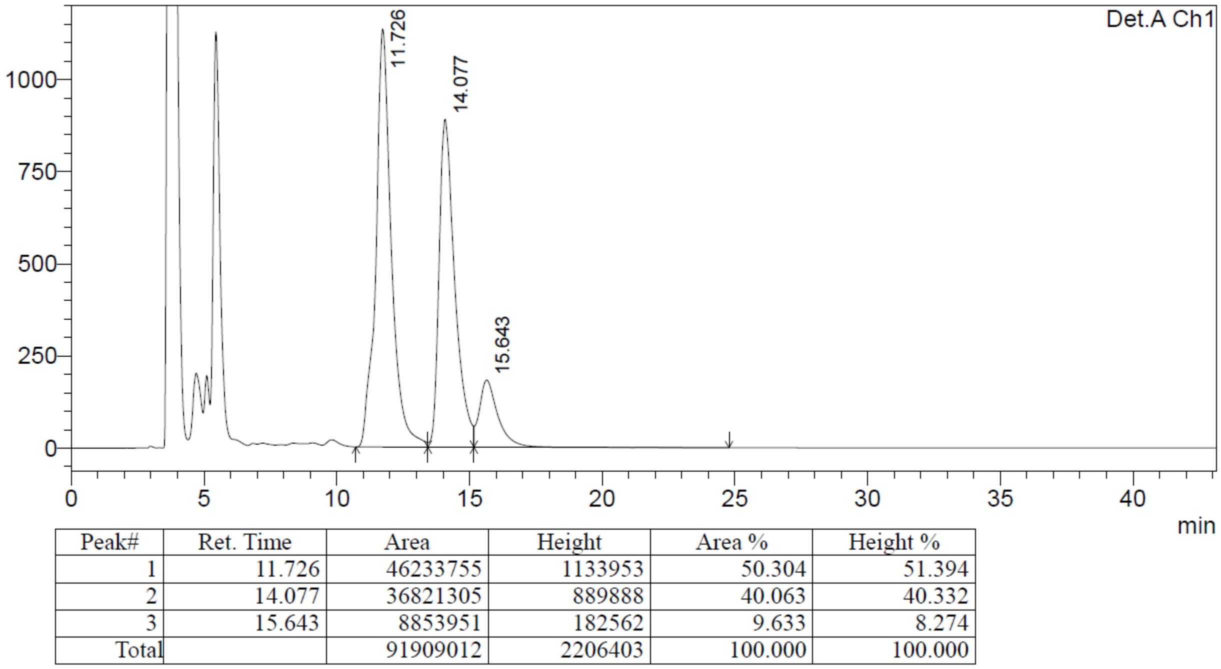

Peak#	Ret. Time	Area	Height	Area %	Height %
1	11.726	46233755	1133953	50.304	51.394
2	14.077	36821305	889888	40.063	40.332
3	15.643	8853951	182562	9.633	8.274
Total		91909012	2206403	100.000	100.000

The *ee* of all samples of compound **5** was determined using chiral HPLC (ADH column, 10% IPA in heptane, flow 1 mL min^{-1}).

Chromatogram of Rac-**5a/5b/5c/5d**

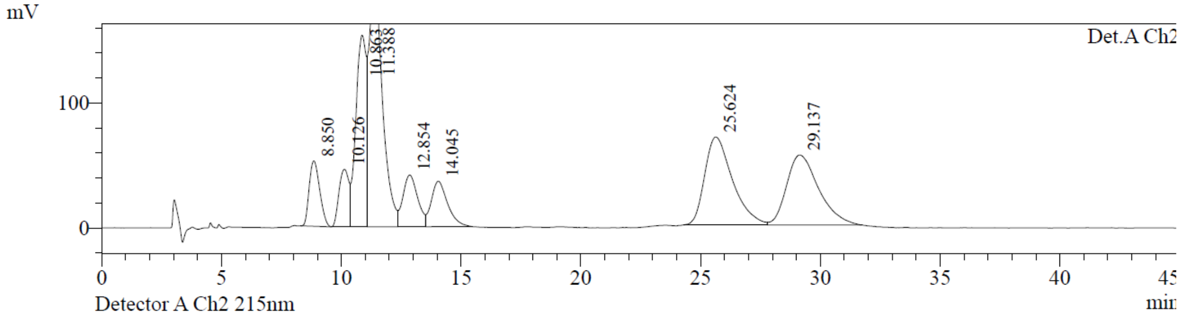

Detector A Ch2 215nm

Peak#	Ret. Time	Area	Height	Area %	Height %
1	8.850	1600253	52326	5.381	7.990
2	10.126	1322410	46043	4.447	7.031
3	10.863	4581356	153235	15.406	23.399
4	11.388	7825259	199320	26.314	30.436
5	12.854	1770198	41340	5.953	6.313
6	14.045	1720010	36305	5.784	5.544
7	25.624	5680413	70329	19.101	10.739
8	29.137	5238349	55990	17.615	8.550
Total		29738248	654889	100.000	100.000

Chromatogram of RR-based-**5a/5b/5c/5d**

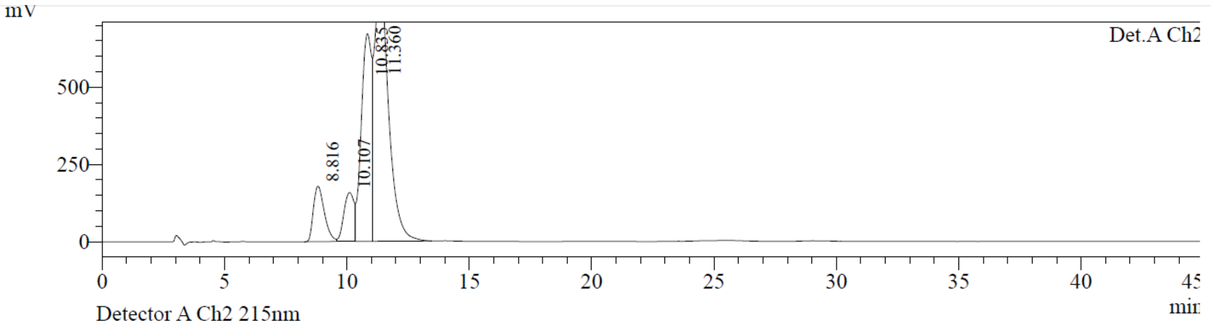

Detector A Ch2 215nm

Peak#	Ret. Time	Area	Height	Area %	Height %
1	8.816	5594536	179079	8.695	9.556
2	10.107	4521248	158359	7.027	8.450
3	10.835	19796649	671702	30.769	35.842
4	11.360	34428043	864913	53.509	46.152
Total		64340476	1874052	100.000	100.000

Chromatogram of SS-based-**5a/5b/5c/5d**

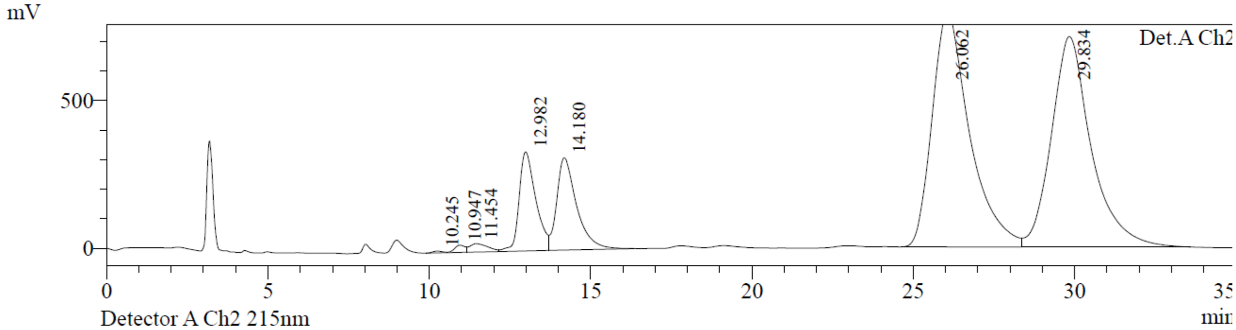

Detector A Ch2 215nm

Peak#	Ret. Time	Area	Height	Area %	Height %
1	10.245	126853	5868	0.083	0.265
2	10.947	552597	24227	0.363	1.093
3	11.454	1103466	28378	0.725	1.281
4	12.982	12071537	334945	7.930	15.116
5	14.180	13371368	312646	8.784	14.109
6	26.062	63910011	798341	41.982	36.028
7	29.834	61094559	711483	40.133	32.108
Total		152230392	2215887	100.000	100.000

7 Determination of absolute configuration

For compound **3**, the absolute configuration of the molecule was assigned based on the elucidation of the structure of a single crystal, which was consequently dissolved and run on the chiral HPLC. It was determined that the second peak corresponded to *SS*-enantiomer.

For compound **2**, the absolute configuration could not be determined using X-ray crystallography. By comparison of the CD spectra, which are near identical for compound **2** and **3**, the first HPLC peak was assigned to the *RR* and the second peak to the *SS*-enantiomer.

7 NMR Spectra

^{1}H NMR spectrum of RS+SR-**2**

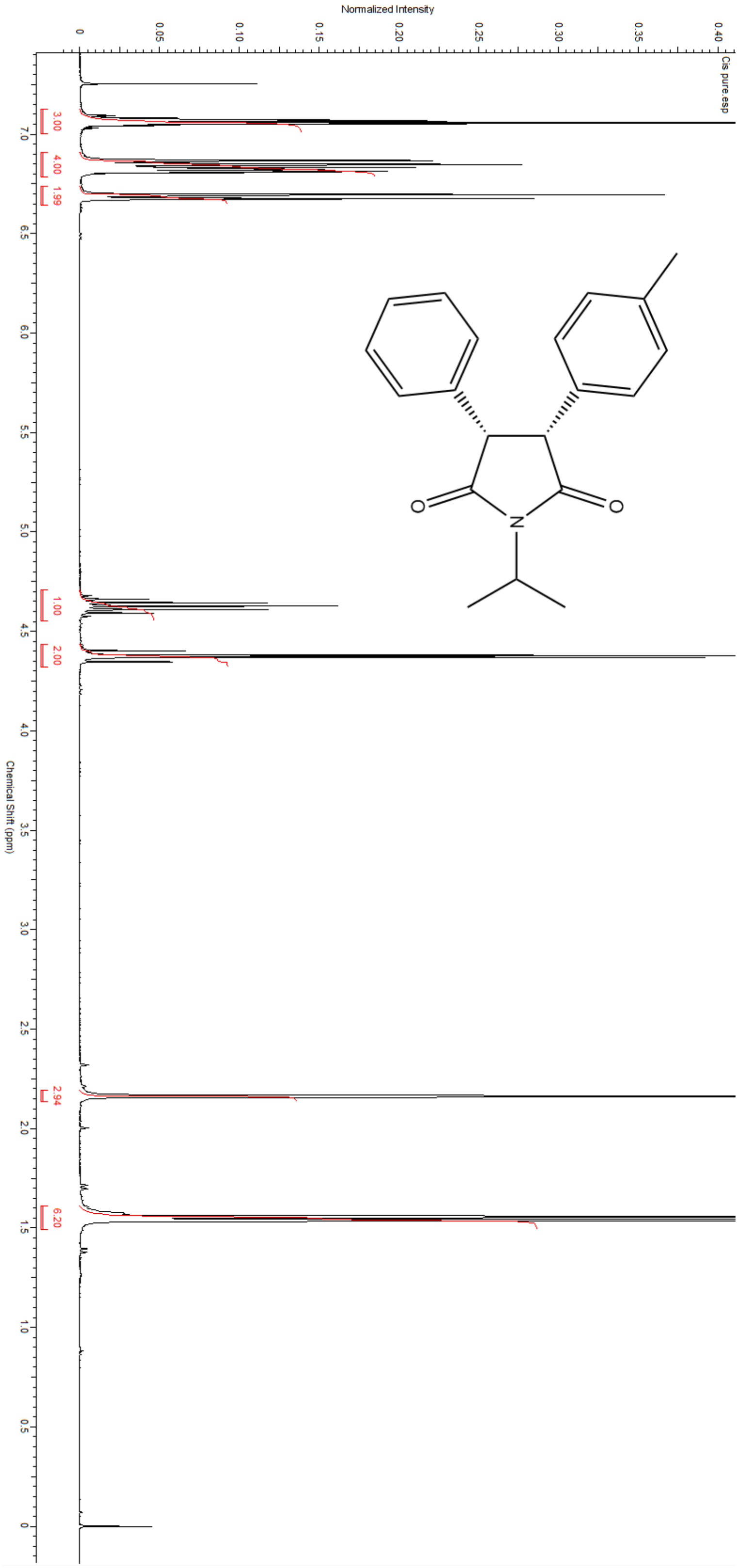

^{1}H NMR spectrum of RR+SS-**2**

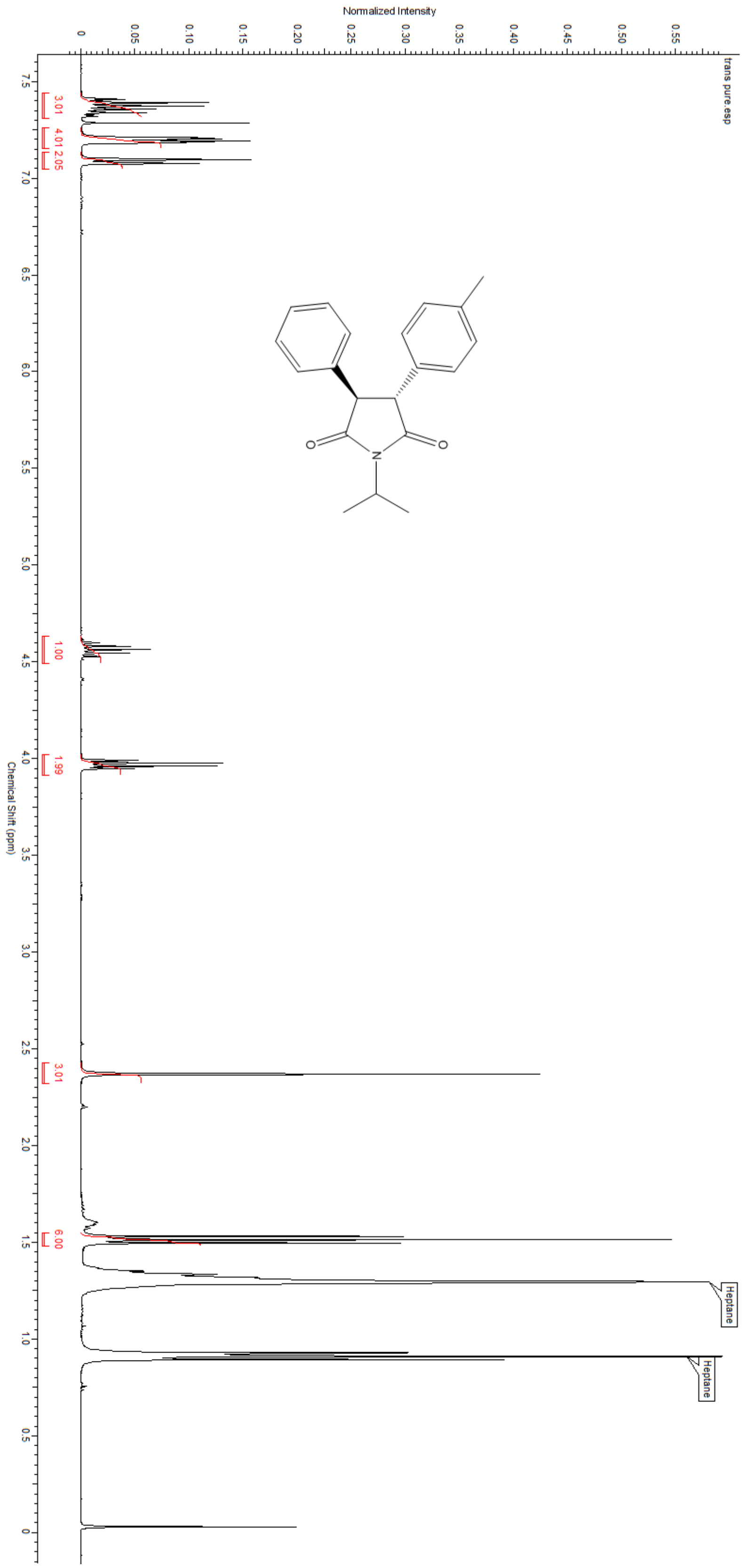

^{1}H NMR spectrum of RR+SS-**3**

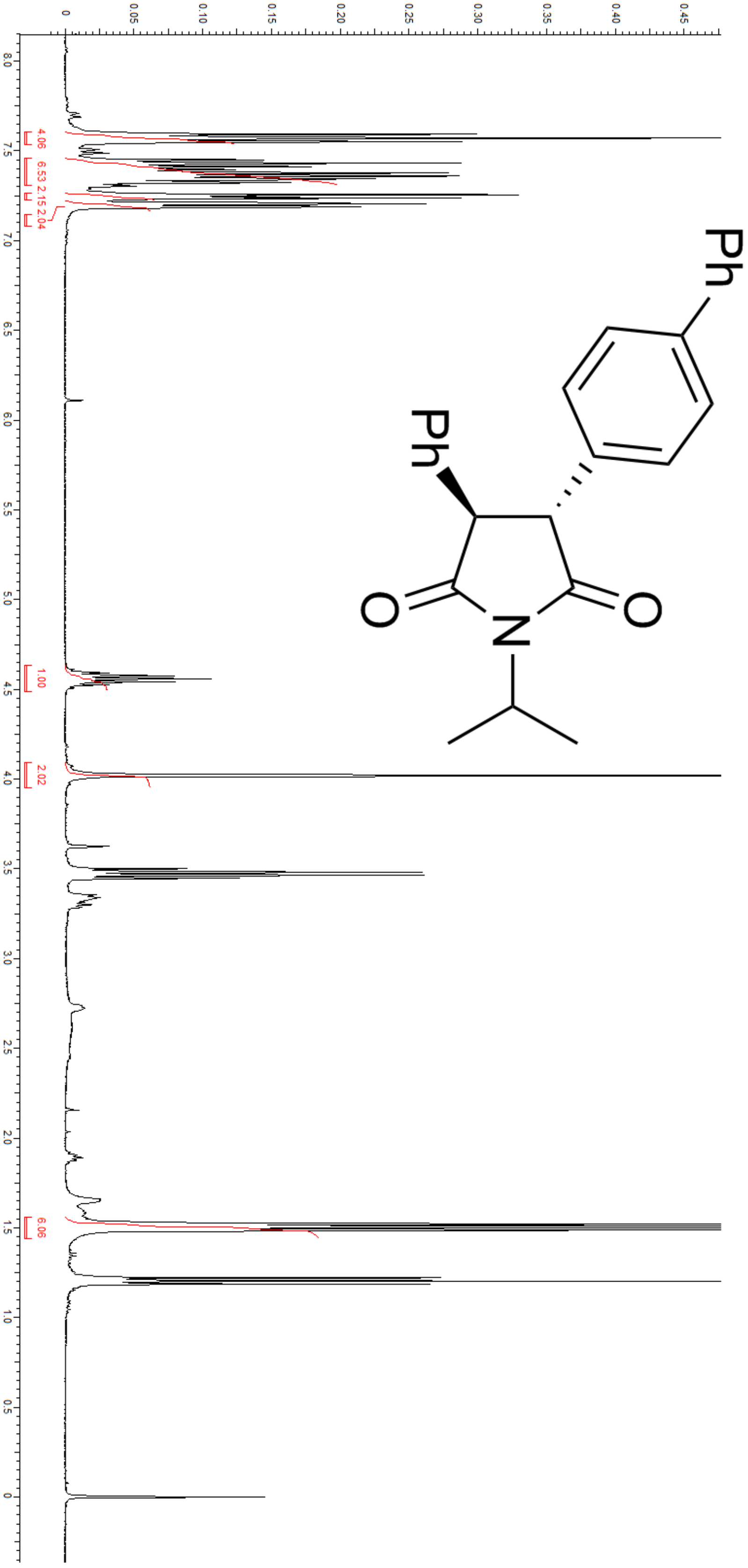

^{1}H NMR spectrum of additives **5a**-**5d**

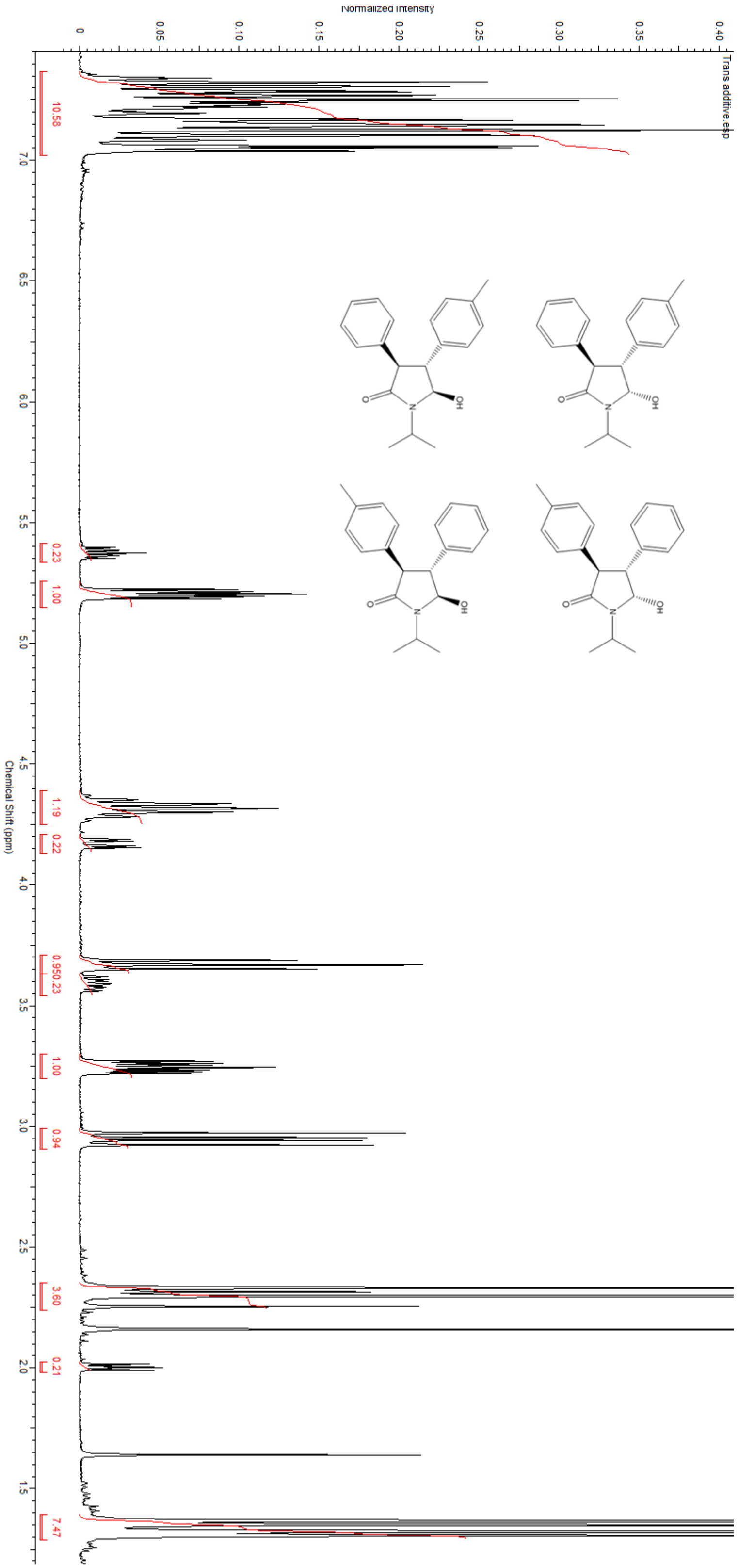
Trans additive.esp
Normalized Intensity
Chemical Shift (ppm)

8 References

[1] S. Hachiya, Y. Kasashima, F. Yagishita, T. Mino, H. Masu, M. Sakamoto, *Chem. Commun.* **2013**, *49*, 4776-4778.
[2] J.B.P.A. Wijnberg, H. E. Schoemaker, W. N. Speckamp, *Tetrahedron* **1978**, *34*, 179-187.
